# Effect of Toxic Components on Microbial Fuel Cell-Polarization Curves and Estimation of the Type of Toxic Inhibition

**DOI:** 10.3390/bios2030255

**Published:** 2012-07-11

**Authors:** Nienke E. Stein, Hubertus V. M. Hamelers, Gerrit van Straten, Karel J. Keesman

**Affiliations:** 1 Wetsus, Centre of Excellence for Sustainable Water Technology, P.O. Box 1113, 8900CC Leeuwarden, The Netherlands; E-Mails: nienke.stein@tno.nl (N.E.S.); bert.hamelers@wetsus.nl (H.V.M.H.); 2 Systems and Control Group, Wageningen University, P.O. Box 17, 6700 AA Wageningen, The Netherlands: E-Mail: Gerrit.vanstraten@wur.nl; 3 Subdepartment of Environmental Technology, Wageningen University, P.O. Box 8129, 6700 EV Wageningen, The Netherlands

**Keywords:** toxicity detection, microbial fuel cell, biosensor, least square estimation, linear regression

## Abstract

Polarization curves are of paramount importance for the detection of toxic components in microbial fuel cell (MFC) based biosensors. In this study, polarization curves were made under non-toxic conditions and under toxic conditions after the addition of various concentrations of nickel, bentazon, sodiumdodecyl sulfate and potassium ferricyanide. The experimental polarization curves show that toxic components have an effect on the electrochemically active bacteria in the cell. (Extended) Butler Volmer Monod (BVM) models were used to describe the polarization curves of the MFC under nontoxic and toxic conditions. It was possible to properly fit the (extended) BVM models using linear regression techniques to the polarization curves and to distinguish between different types of kinetic inhibitions. For each of the toxic components, the value of the kinetic inhibition constant Ki was also estimated from the experimental data. The value of Ki indicates the sensitivity of the sensor for a specific component and thus can be used for the selection of the biosensor for a toxic component.

## 1. Introduction

Electrochemically active microorganisms in a microbial fuel cell (MFC) oxidize organic material to carbon dioxide, protons and electrons. In the absence of a soluble electron acceptor they donate the electron to a solid electron acceptor, the anode. An electron acceptor at the second electrode, the cathode, is reduced so that the electrons flow through the electrical circuit and thus produce an electrical current. The rate of degradation of organic material is proportional to the current. Therefore, the current is a measure for the metabolic activity of the bacteria in the microbial fuel cell. 

The MFC has many applications such as a renewable source for energy production, bio-electrochemical production of chemicals or as a BOD sensor in water [1,2,3,4]. This paper, however, focuses on the use of an MFC as sensor for toxic chemicals in water. 

The MFC produces a constant current under constant conditions. However, when a toxic component enters the anodic compartment of the MFC, the bacteria are affected by the toxic component, resulting in a change of current. This change is often seen as a decrease in current [2,5]. The MFC can therefore be used as a senor for toxic components in water.

In research on MFCs, the cells are typically characterized by polarization curves [6,7,8]. When the anode potential is varied, the resulting current gives information related to the energetic losses in the system and the metabolic state of the bacteria. At a certain potential the current will be zero. This zero-current potential mainly depends on the oxidation potential of the organic material, the substrate. The difference between this zero-current potential and the actual anode potential is called the anodic overpotential. This anodic overpotential determines the energy that bacteria can gain from the oxidation of the substrate and the corresponding donation of electrons to the anode. A polarization curve thus shows the dependency of the current on the anodic overpotential. 

The Butler Volmer Monod (BVM) model was developed to describe polarization curves of the MFC under nontoxic conditions [9]. Under toxic conditions one of the extended BVM models can be used [10]. These models are based on biochemical and electrochemical kinetics. The extended BVM models also include enzyme inhibition kinetics. Theoretically, it is possible to distinguish between four types of toxic inhibitions. 

(1) Overall inhibition of the bacteria, where the toxic component acts as an irreversible inhibitor. This gives the following model

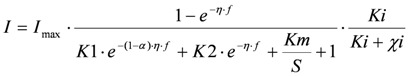
(1)

In the following, this model is referred to as “Itox”.

(2) Inhibition effect on the ratio between electrochemical and biochemical rate constant. This gives the following model:

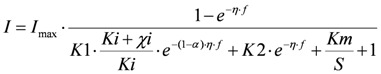
(2)
where, for example, the toxic component acts as an electron acceptor. This model is referred to as “IK1”.

(3) Inhibition effect on the ratio between the forward and backward reaction rate constant of substrate oxidation. This gives the following model:

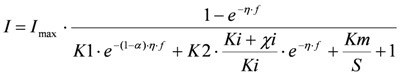
(3)
and is referred to as ‘IK2’

(4) Competition between substrate and toxic component to bind to the redox complex. This gives

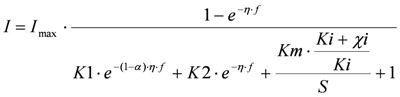
(4)
and is referred to as ‘IKm’. 

In these models *I* (mA) is the current, *I*_max_ (mA) is the maximum current determined by maximum enzymatic rates of microorganisms, *η *(V) is the overpotential, *K*1 (−) is a lumped parameter describing the ratio between biochemical and electrochemical rate constants, *K*2 (−) is a lumped parameter describing the forward over backward biochemical rate constants, *Km* (mol/L) is the substrate affinity constant, and *S* (mol/L) the substrate concentration. Furthermore, f = F/RT with F (C/mol) being the Faraday’s constant, R (J/mol/K) the gas constant and T (K) temperature. *Ki* is the inhibition constant of component *i* and *χi* is the concentration of toxic component *i*. 

*Ki* is the inhibition constant that shows how toxic the component is for the bacteria and thus how sensitive the sensor is for the toxic component. Hence, a low value for *Ki* gives a very sensitive sensor. For each of these inhibition mechanisms the polarization curves look different. Furthermore, for each of the mechanisms it is possible to determine at which overpotential the current changes most when a toxic component enters the cell [10]. As yet, no experimental data on polarization curves in the presence of toxic components are available in the literature. In this study, we investigate whether it is possible to fit one of the models (1–4) to the polarization curves when toxic components are present in the system. Furthermore, we study if it is possible to distinguish between different types of toxic components based on the different enzyme inhibition kinetics. 

First, polarization curves under non-toxic and toxic condition using four components at three different concentrations were experimentally determined. These polarization curves were then compared with the model responses (1–4), describing the four types of toxic inhibitions. 

## 2. Experimental Section

### 2.1. Experiments

Two-chamber microbial fuel cells using graphite plate electrodes were constructed as described in Heijne *et al.* [6]. The cells could each be controlled individually, both mechanically and electrically. A mixed culture of microorganisms was grown in a single MFC with 20 mL of anolyte taken from an active microbial fuel cell and a biofilm formed on the anode at a set anode potential of −0.4 V *vs.* Ag/AgCl. The medium was used as described in Stein *et al.* [5] using 5 mM acetate as substrate. The medium was purged with nitrogen to keep it anaerobic and the continuous flow rate was 0.7 mL/min. The microbial fuel cells were operated at a constant anode potential of 0.3 V. For polarization curves, the anode potential was increased stepwise by 0.025 V every ten minutes from −0.4 V to −0.15 V. The current was measured every ten seconds. The average current of the last 10 data points per potential was calculated and used in the estimation procedure. Open circuit potential was measured approximately two hours after the polarization curve was made. 

To measure the influence of toxic components, the component was added to the medium and the medium was continuously supplied at least two hours (>3 HRT) before the polarization curve was made. The following components were added, one for each experiment: nickelchloride (10, 20, 30 mg/L nickel), sodiumdodecylsulfate (SDS) (10, 25, 50 mg/L), bentazon (1 and 3 mg/L) and potassium ferricyanide (0.5, 1, 2 mM). These four components were chosen, because they are very different types of toxic components. Nickel is a heavy metal, SDS is used in soaps as a surfactant, bentazon is a herbicide acting on photosynthetic activity and ferricyanide is very fast electron acceptor. The concentrations were such that a change in polarization curve could be observed. The sensor was not optimized for sensitivity yet. The concentrations may therefore seem rather high compared to e.g., surface water concentrations.

### 2.2. Estimating the Type of Kinetic Inhibition

Given the experimental data of the polarization curves, the extended BVM models (1–4) were subsequently fitted to the data to determine the values of kinetic parameters *K*1, *K*2 and *Km* and the type of toxicity. The curve under clean conditions was used to determine the values of *K*1, *K*2 and *Km* using linear regression techniques. The value of *Ki* was determined from the experiments with addition of toxic components. In these experiments the concentration of the toxic component *χi* was considered to be known, as bulk concentrations in the cell were measured. The best fit was determined for each type of toxicity and the value for *Ki* was calculated using the known concentration of toxic component. The best fit indicates which type of inhibition is caused by the supplied toxic component. The calculations are presented in Appendix 1. 

## 3. Results and Discussion

### 3.1. Effect of Toxic Components on Polarization Curves

Polarization curves were made under clean conditions and under conditions when a toxic component was present in the sensor. Through these experiments we investigated in a systematic way what the effect of toxic components, such as nickel chloride (nickel), sodium dodecyl sulfate (SDS), bentazon and potassium ferricyanide, is on the current at different overpotentials. The effect of the concentration was also investigated. 

**Figure 1:**
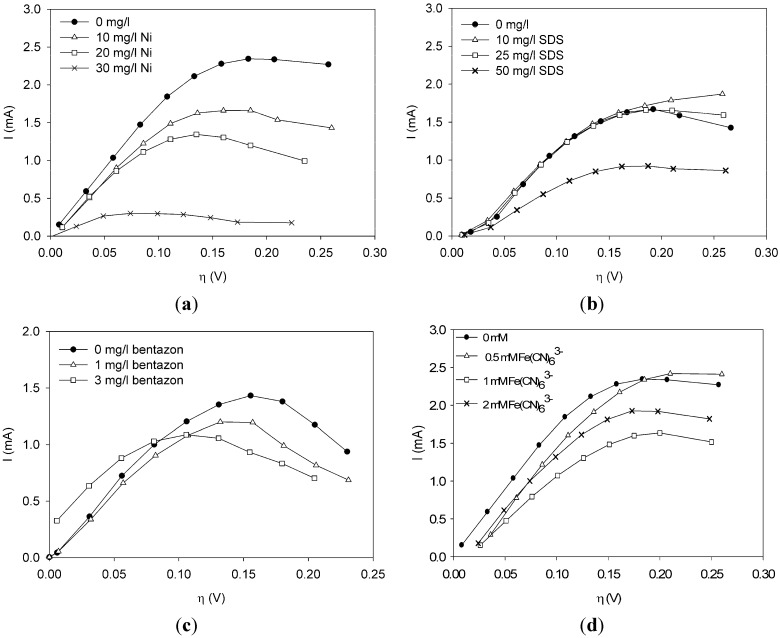
Effect of chemical components on the current (**a**) nickel. (**b**) sodium dodecyl sulfate (SDS). (**c**) bentazon. (**d**) potassium ferricyanide.

As a first toxic component, nickel was dosed at three concentrations: 10, 20 and 30 mg/L. Data are shown in Figure 1(a). Comparing the polarization curve when nickel is present with the curve under non-toxic conditions, the curve shifts down when nickel is present in the sensor. The higher the concentration, the larger the shift. Hence, there seems to be a dose-response relationship of current change to nickel concentration. This was already found from experiments in the MFC-based biosensor at constant anode potential [5], but it is thus also found from the polarization curves. 

Figure 1(b) shows the effect of SDS on the current. Notice from Figure 1(b) that a concentration of 10 or 25 mg/L SDS does not show a strong effect on the metabolic activity of the bacteria. The polarization curve is more or less the same as under non-toxic conditions. When the concentration is increased to 50 mg/L, however, the curve shifts down and a lower current is observed at all overpotentials. 

Bentazon was added to the sensor at a concentration of 1 and 3 mg/L. Notice from Figure 1(c) that at all overpotentials the polarization curve with 1 mg/L bentazon is lower than the polarization curve without any toxic components. The curve related to 3 mg/L bentazon has shifted to the left and reaches a lower maximum as compared to the curve without any bentazon present. Furthermore, the current related to 3 mg/L bentanon is higher at low overpotentials and lower at overpotentials above 0.1 V. 

Ferricyanide is a negative ion complex (FeCN_6_^3^^−^) that can take up one electron to form ferrocyanide (FeCN_6_^4^^−^). Ferri/ferrocyanide is known to be a very fast electron couple. It is frequently used at the cathode in research on MFCs to prevent the electron uptake being the rate limiting step [1,11]. When ferricyanide is present in the anode compartment, it is expected that it will take up electrons from the bacteria. Hence, it could act as an electron shuttle or as the final electron acceptor. If it acts as an electron shuttle, it takes up electrons from the bacteria, forming ferrocyanide, and then donates the electron to the anode forming ferricyanide again. The standard electron potential of the couple FeCN_6_^3^^−^/FeCN_6_^4^^−^ is E_0_ = +0.14 V *vs.* an Ag/AgCl reference electrode. The equilibrium potential of the bacteria is around −0.46 V *vs.* Ag/AgCl. The bacteria can thus donate the electron to ferricyanide, which is then oxidized. When making the polarization curve, the anode potential is varied between −0.45 V and −0.150 V and thus ferrocyanide can be reduced at the anode for the whole range of the polarization curve. Ferricyanide is used in several applications as electron shuttle/mediator (e.g., in a BOD sensor [12,13]) and is considered nearly non-toxic [12,14]. It also happens that some of the oxidized form is washed out of the compartment before it is reduced at the anode. Then, it acts as a final electron acceptor in the MFC and thus lowers the measured current. 

Ferricyanide was dosed to the sensor at concentrations of 0.5 mM, 1 mM and 2 mM. As can be seen from Figure 1(d), the current decreased due to the presence of ferricyanide. The decrease of current was larger when 1 mM was dosed than when 2 mM was dosed. The polarization curve when 0.5 mM was present is lower for overpotentials below 0.175 V than the polarization curve when no toxic component was present. 

### 3.2. Estimation of Type of Toxic Inhibition

#### 3.2.1. Fitting Nickel

First, the BVM model (*χi* = 0) was fitted to the polarization curve without toxic component. The following estimates (denoted by ^) for *K*1, *K*2 and *Km* were found using weighted least squares techniques [15]:

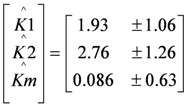


Given the values of 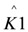 and 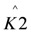 the models were then fitted to the polarization curve related to the case with 20 mg/L nickel in the sensor. The value of *Km* could not be estimated accurately because of 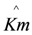
~0.1 mM and *S* = 5 mM, *S* >> *Km* and leading to *Km*/*S* << 1 in Equation (1). Moderate toxicity levels such that 
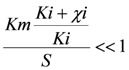
 fiting the data to model IKm (Equation (4)) will result in inaccurate estimates of *Ki* and are thus not considered here. For the three remaining types of toxic inhibition, the values as shown in Table 1 were found: 

**Table 1:** Fitting of the polarization curve for 20 mg/L Ni to the extended Butler Volmer Monod (BVM) models (Equations (1)–(3)).

*Toxic inhibition*	*Ki (mg/L)*	*J_N_ (mA^2^)*
Itox	31.68	0.30
IK1	14.72	3.53
IK2	14.87	3.70

**Figure 2:**
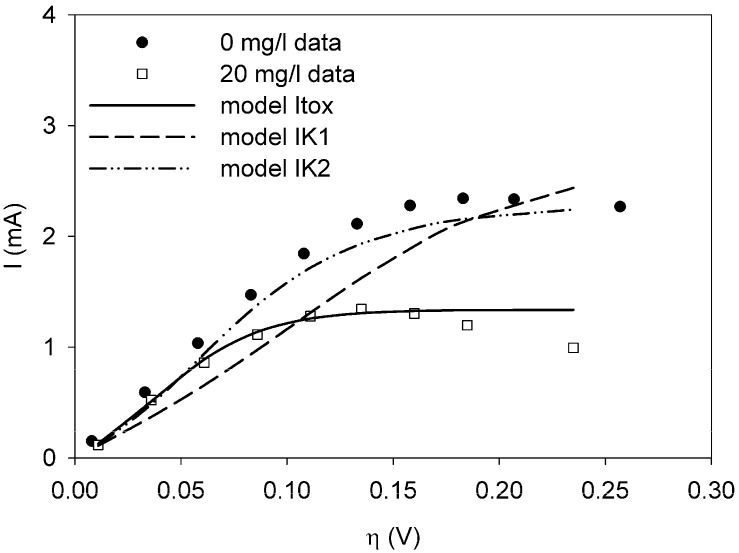
Polarization curves for a clean sensor (dots) and with 20 mg/L Ni present (squares). The data for 20 mg/L Ni were used to fit to model Itox (solid line), IK1 (dash), and IK2 (dot-dash-dot) using weighted least squares techniques.

The sum of squared errors (*J_N_*) is a measure for the quality of the fit. A smaller value of *J_N_* indicates a better fit. Hence, in Table 1 model Itox gives the best result. The fits are shown in Figure 2. The fits for model IK1 and IK2 are not very good, while the fit for model Itox is much better. 

When the model is fit to the data for 10 mg/L nickel, a similar result is found. The sum of squared errors is lowest for model Itox and the estimated value for Ki is similar, 30.1 mg/L. With 30 mg/L nickel in the sensor, the model Itox also gives the best fit. The value for Ki is estimated to be 4.57 mg/L. All results can be found in Table S1A, Table S1B and Table S1C in Appendix 2. 

#### 3.2.2. Fitting SDS

As at low concentrations of SDS (10, 25 mg/L) no toxic effect on the polarization curves was seen, the three models were only fitted to the polarization curve made with 50 mg/L SDS in the sensor (data in Figure 1(b)). The best fit was obtained for model IK1 but this gave a negative value for *Ki* which could not be interpreted physically. The fit for model IK2 was not significantly worse, only by a factor 1.5. The fit for model Itox was only two times worse than for model IK1. In this case there was no significant difference between the fit of the three models using weighted least squares estimation, and it was therefore hard to say according to which inhibition type SDS acted on the bacteria. For model Itox, the calculated value for *Ki* was equal to 85.37 mg/L while for model IK2 this was 49.60 mg/L. This explains the very small change at lower concentrations of SDS in the sensor. Hence, the bacteria are not very sensitive to SDS. The calculations for all models can be found in Table S2 in Appendix 2. 

#### 3.2.3. Fitting Bentazon

Fitting the models to the data in Figure 1(c), model Itox gave the best fit. When 1 mg/L was dosed, the difference between the sums of squared errors (*J_N_*) between the three models was not very large. Model Itox gave a *J_N_* of 0.32 mA^2^ with *Ki* 3.0 mg/L. When 3 mg/L was dosed, it was clear that model Itox fitted best with a sum of squared errors of 0.63 mA^2^. The calculations for all models can be found in Table S3A, Table S3B in the Appendix 2.

#### 3.2.4. Fitting Potassium Ferricyanide

Polarization curves with ferricyanide present in the sensor were compared to the curve when no toxic component was present. The sum of squared errors *J_N_* was smallest for model IK2 when 0.5 mM FeCN_6_^3−^ was present, while *J_N_* was smallest for model Itox when 1 or 2 mM FeCN_6_^3−^ was dosed (see Table 2). With all three concentrations, model IK1 did not have the smallest sum of squared errors. 

**Table 2:** Fitting of the polarization curve for 0.5 and 2 mM FeCN_6_^3^^−^ to the extended BVM models (Equations (1)–(3)).

*Toxic inhibition*	*Ki (mM)*	*J_N_ (mA^2^)*
Itox (0.5 mM)	48.44	0.46
**IK2 (0.5 mM)**	**0.11**	**0.12**
**Itox (2 mM)**	**10.26**	**0.11**
**IK2 (2 mM)**	0.51	0.39

**Figure 3:**
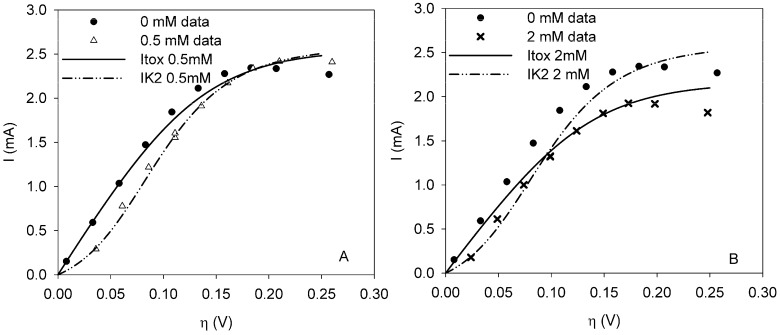
Polarization curves under clean conditions (dot) and when ferricyanide is present in the sensor at a dosage of (**a**) 0.5 mM (trangle) or (**b**) 2 mM (cross). Model Itox (solid line) and model IK2 (dot-dash-dot) are fitted to data from the curves with ferricyanide.

At 0.5 mM FeCN_6_^3^^−^ the fit of model IK2 is 3.7 times better than model Itox. When the concentration is increased, model Itox fits better. The factor is more or less the same. This means that model IK2 does not fit significantly better at 0.5 mM than model Itox at 2 mM. Thus as the fits are equally good, there is a change in the mode of inhibition of ferricyanide with increasing concentration. Figure 3 shows the fit of model Itox and IK2 for the polarization curves with FeCN_6_^3^^−^. 

If ferricyanide would act as an electron shuttle, it is expected to inhibit the bacteria in the sensor according to model IK1, because parameter K1 describes the ratio between the electrochemical and biochemical rate constants. An electron shuttle could affect this ratio, because part of the redox complex is not fully oxidized at the anode but partly at the toxic component and hence the current decreases. However, this was not found from the analysis of the polarization curves. For a comparison with the data in Table 2, the sum of squared errors for IK1 is 0.65 mA^2^ at 0.5 mM and 1.09 mA^2^ at 2 mM. The calculated values for all models can be found in Table S4A, Table S4B and Table S4C in Appendix 2.

#### 3.2.5. Comparing the Effects of Toxic Components

For nickel, SDS and bentazon, it is found that model Itox gives the best fit. Ferricyanide fits best to model IK2 or Itox, depending on the concentration. It thus seems that we can distinguish between different types of toxicity for different components. Measuring more components will result in a better overview for different types of toxicity. The results show the effect of single components. Mixes of potentially toxic components may give a different effect on the polarization curve and therefore on the estimated type of toxic inhibition. In total nine polarization curves of the MFC biosensor containing (toxic) components were fitted to the extended BVM models. Seven of these polarization curves fitted best to model Itox (Equation (1)) while one (FeCN_6_^3^^−^ 0.5 mM) fitted best to model IK2 and one could not be clearly distinguished (SDS, 50 mg/L). The models are based on enzyme inhibition kinetics while combined effects may also play a role. In practice it may not be just one type of inhibition. Combinations of inhibitions may be possible as well. The concentration of the toxic component may also play a role in determining the type of inhibition. This has not been taken into account in this study, as the model set contains models that already can give good fits in most experiments.

## 4. Conclusions

This paper shows for the first time the effect of toxic components on polarization curves in an MFC-based biosensor. From experimentally determined polarization curves, it is clear that toxic components have an effect on the electrochemically active bacteria in the cell, which is seen as a shift in the polarization curve. The way the polarization curve changes depends on the type of components and their concentration. There is a dose-response relationship for all the tested components, meaning that current decreases more when the concentration of toxic component is higher. This paper shows that dose-response relationships can be visualized when polarization curves are made. As seen for nickel and SDS, a higher concentration leads to a lower current at all overpotentials. 

It was possible to fit the extended BVM models (1–4) to the polarization curves related to all the components used. Comparing the sum of squared errors for each fit, it is clear that the model Itox fits best for nickel and SDS and bentazon. For potassiumferricyanide it seems that combined effects play a role. At low concentrations, only one type of kinetic inhibition plays a role (model IK2). When the concentration increases, however, model Itox, representing the overall inhibition of the cell, gives a better fit. 

The results show that the extended BVM model can be used to distinguish between different types of kinetic inhibitions although in most cases the same type of inhibition was observed, *i.e.*, the inhibition of the whole organisms (model Itox). The extended BVM-model can determine the type of kinetic inhibition of a component, but the model may need to be improved for combined effects or for a concentration effect. 

The value of the inhibition constant Ki can be determined. The value of Ki tells something about the sensitivity of the sensor for the measured component. This is important to know when deciding whether the sensor is suitable for a specific application. 

## Appendix 1

This fitting can be done by using linear regression techniques [16,17]. Notice that the BVM-model can be written linear in its kinetic parameters [18,19], as

(5)

After writing Equation (5) in the general linear regression form

(6)

in which the error term e is added to represent measurement and modelling errors, the ordinary least squares estimate  is given by

(7)

with  the estimate of [*K*1 *K*2 *Km*]^T^. The Nx3-dimensional regressor matrix

contains the N values of the overpotential at which the current is measured and the estimated substrate concentration S in the biofilm.

containing the measured current (*I**) at each overpotential.

In what follows, S is assumed to be constant. S can be estimated online from measurements of the electrical current (not shown here). Alternatively, the concentration can also be measured in the bulk of the sensor under the assumption that the substrate concentration in the bulk and in the biofilm are equal.

In what follows, a weighted least squares, is used, where the estimate is given by:

(8)

with W the weighing matrix (for details see [15]).

Subsequently, the four extended BVM models (1–4) were then fitted to the curves with toxic component, using the estimates of *K*1, *K*2 and *Km* obtained from the clean curve. The value of *Ki *could be estimated, as in the experiments the concentration of toxic component *χi* was known. The best fit was determined for each type of toxicity and the value for *Ki* was calculated. However, this best fit is not equally good for each of the four types of toxicity, resulting into different values for the sum of squared errors (*J_N_*):

(9)

with *N* the number of measurements,  the k-th measurement of the current and  the estimated current at measurement index *k* using the estimated parameter values of *K*1, *K*2 and *Km*. Index *j* denotes the model type (Itox, IK1, IK2, or IKm) with the corresponding equation to calculate the estimated current.

For example, for model Itox this equation looks as follows:

(10)

A smaller *J_N_* implies a better fit to the data.

## Appendix 2

The Tables below show the fitting results of the three models Itox (Equation (1)), IK1 (Equation (2)) and IK2 (Equation (3)) to the experimental data. The polarization curve of the clean sensor was used to estimate *K*1 and *K*2 in Equations (1)–(4). The polarization curve related to the experiments with a toxic component, using the known concentration, was subsequently used for the estimation of *Ki*, the inhibition constant, for the estimation of constants a weighted least squares (WLS) method was applied, as described by Stein *et al.* [15].

### S2.1.Nickel

**Table S1A:** Nickel 10 mg/L.

*Toxic inhibition*	*Ki (mg/L)*	*J_N_ (mA^2^)*
**Itox**	**30.31**	**0.15**
IK1	7.62	1.97
IK2	7.43	1.81

**Table S1B:** Nickel 20 mg/L.

*Toxic inhibition*	*Ki (mgl)*	*J_N_ (mA^2^)*
**Itox**	**31.68**	**0.30**
IK1	14.72	3.53
IK2	14.87	3.70

**Table S1C:** Nickel 30 mg/L.

*Toxic inhibition*	*Ki (mg/L)*	*J_N_ (mA^2^)*
**Itox**	**4.57**	**0.07**
IK1	9.02	8.70
IK2	24.42	16.26

### S2.2. Sodium Dodecyl Sulfate (SDS)

**Table S2:** SDS 50 mg/L.

*Toxic inhibition*	*Ki (mg/L)*	*J_N_ (mA^2^)*
Itox	85.37	1.49
**IK1**	**−7.82**	**0.71**
IK2	49.60	1.07

### S2.3. Bentazon

**Table S3A:** Bentazon 1 mg/L.

*Toxic inhibition*	*Ki (mg/L)*	*J_N_ (mA^2^)*
**Itox**	**3.00**	**0.32**
IK1	0.51	1.03
IK2	13.29	0.35

**Table S3B:** Bentazon 3 mg/L.

*Toxic inhibition*	*Ki (mg/L)*	*J_N_ (mA^2^)*
**Itox**	**9.11**	**0.63**
IK1	−0.15	15.11
IK2	−2.95	1.44

### S2.4. Ferricyanide

**Table S4A:** Ferricyanide 0.5 mM.

*Toxic inhibition*	*Ki (mM)*	*J_N_ (mA^2^)*
Itox	48.44	0.46
IK1	0.19	0.65
**IK2**	**0.11**	**0.12**

**Table S4B:** Ferricyanide 1 mM.

*Toxic inhibition*	*Ki (mM)*	*J_N_ (mA^2^)*
**Itox**	**2.18**	**0.12**
IK1	0.22	0.45
IK2	0.16	1.61

**Table S4C:** Ferricyanide 2 mM.

*Toxic inhibition*	*Ki (mM)*	*J_N_ (mA^2^)*
**Itox**	**10.26**	**0.11**
IK1	0.71	1.09
IK2	0.51	0.39
